# Do low-carbohydrate diets increase energy expenditure?

**DOI:** 10.1038/s41366-019-0456-3

**Published:** 2019-09-23

**Authors:** Kevin D. Hall, Juen Guo, John R. Speakman

**Affiliations:** 1 National Institute of Diabetes and Digestive and Kidney Diseases, Bethesda, MD 20892 USA; 2 Institute of Biological and Environmental Sciences, University of Aberdeen, Aberdeen, UK; 3 Institute of Genetics and Developmental Biology, Chinese Academy of Sciences, Beijing, China

**Keywords:** Metabolism, Clinical trial design

Proponents of low-carbohydrate diets have claimed that such diets result in a substantial increase in total energy expenditure (TEE) amounting to 400–600 kcal/day [1] thereby providing patients with a “high calorie way to stay thin forever” [2]. However, a recent systematic review and meta-analysis of controlled feeding studies found no meaningful TEE effects comparing isocaloric diets with equivalent amounts of protein but varying in their proportion of carbohydrate to fat [3]. Nevertheless, it is possible that these past studies may have failed to create the appropriate conditions to reveal the hypothesized increase in TEE during a low-carbohydrate diet [4].

For example, low-carbohydrate diets may attenuate the usual reduction in TEE that occurs after weight loss. In support of this possibility, Ebbeling et al. recently reported that a low-carbohydrate diet significantly increased TEE by ~200–300 kcal/day compared with a high-carbohydrate diet during maintenance of lost weight [5]. Here, we critically examine this conclusion by reanalyzing the data of Ebbeling et al. to investigate whether the reported diet differences in TEE were commensurate with other measurements, robust to various assumptions, and obeyed the physical law of energy conservation.

## Reported results were not calculated according to the preregistered statistical analysis plan

The primary outcome of the study by Ebbeling et al. was to compare TEE between low-, moderate-, and high-carbohydrate diets using the doubly labeled water (DLW) method. The original preregistered protocol and analysis plan addressed whether the *reduction* in TEE during maintenance of lost weight depended on the dietary carbohydrate to fat ratio *when compared with the pre-weight loss baseline*—a design similar to a pilot study that was used to power the study in question [6].

Reporting study outcomes according to prespecified analysis plans helps reduce bias [7, 8]. While the original preregistered analysis plan was in place for most of the study’s history, it was changed after all subjects had completed the trial. Rather than comparing TEE during weight loss maintenance, the revised analysis plan used the immediate post-weight loss period as the anchor point. Unfortunately, the pilot study did not measure TEE in the period immediately post-weight loss and therefore no appropriate data were available to inform the power calculations for the revised analysis plan and Ebbeling et al. did not report the results of the originally planned analysis in the final published paper [5]. We downloaded the individual subject data and SAS statistical analysis code from the Open Science Framework website (https://osf.io/rvbuy/) and reanalyzed the data according to the original preregistered analysis plan.

## The original preregistered analysis plan did not result in significant diet differences in TEE

**Fig. 1:**
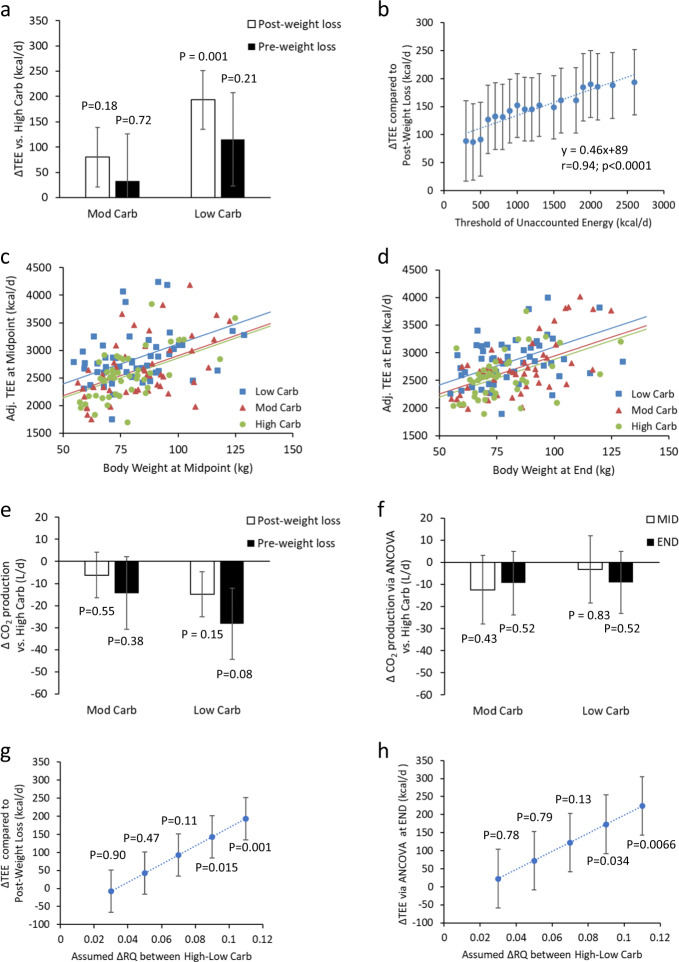
**a** Intention to treat analysis of differences in total energy expenditure (TEE) consuming low- and moderate-carbohydrate diets compared with subjects consuming a high-carbohydrate diet. The open bars illustrate the significant effect of the low-carbohydrate diet on average TEE during weight loss maintenance as compared with the immediate post-weight loss period according to the revised analysis plan. The black bars indicate the lack of significant effect of diet on average TEE during weight loss maintenance as compared with the pre-weight loss baseline period according to the original analysis plan. **b** Difference in TEE between low- and high-carbohydrate diets (calculated using the revised plan comparing with the post-weight loss TEE) as a function of the threshold used to filter out subjects with excessive relative amounts of unaccounted energy. The rightmost data point includes all 162 subjects with as much as 2600 kcal/day of unaccounted energy and corresponds to the diet effect size reported by Ebbeling et al. according to their revised analysis plan. The leftmost data point indicates a greatly reduced effect size and includes 81 subjects with as much as 300 kcal/day of unaccounted energy. **c** Exploratory ANCOVA analysis of TEE adjusted for age, sex, and height plotted against body weight at the midpoint and **d** end of the weight loss maintenance period. The lines are the best-fit lines with common slope for low-, moderate-, and high-carbohydrate diets. **e** Differences in daily average CO_2_ production comparing low- and moderate-carbohydrate diets with the high-carbohydrate diet. No significant effects of diet were observed regardless of whether the measurements during weight loss maintenance were compared with the pre-weight loss baseline period (black bars) or compared with the immediate post-weight loss period (open bars). **f** Differences in daily average CO_2_ production comparing low- and moderate-carbohydrate diets with the high-carbohydrate diet at the middle (open bars) and at the end (black bars) of the study as estimated by ANCOVA. **g** Difference in TEE between low- and high-carbohydrate diets (calculated using the revised plan comparing with the post-weight loss TEE) as a function of the assumed differences in daily RQ between the high- and low-carbohydrate diets. **h** Difference in TEE between low- and high-carbohydrate diets at the end of the study as estimated by ANCOVA as a function of the assumed differences in daily RQ between the high- and low-carbohydrate diets. Error bars are ± SE (Color figure online)

In the intention to treat analysis, the low-, moderate-, and high-carbohydrate groups decreased TEE by 240 ± 64 kcal/day, 322 ± 66 kcal/day, and 356 ± 67 kcal/day compared with the pre-weight loss baseline period, respectively (*p* = 0.43 for the test of equivalence between the diets). Pairwise comparisons of TEE diet differences with respect to the pre-weight loss baseline were not significant between diets (Fig. 1a).

In the so-called “per-protocol group” whose weights remained within ±2 kg during the weight maintenance phase, pairwise TEE comparisons with respect to the pre-weight loss baseline were not significant between diets (*p* > 0.35). The low-, moderate-, and high-carbohydrate groups decreased TEE by (mean ± SE) 262 ± 72 kcal/day, 254 ± 75 kcal/day, and 356 ± 80 kcal/day, respectively, compared with the pre-weight loss baseline period (*p* = 0.59 for the test of equivalence between the diets).

## Reported diet differences were uncorroborated by measures of various components of TEE

Whereas the TEE differences between low- and high-carbohydrate diets reported by Ebbeling et al. were substantial and statistically significant when analyzed according to the revised plan, the various components of TEE were not significantly different and failed to corroborate the reported TEE differences. In particular, differences between the low- and high-carbohydrate diets in resting energy expenditure (0.2 ± 17 kcal/day; *p* = 0.99), total physical activity (19,394 ± 17,800 counts/day; *p* = 0.28), moderate to vigorous physical activity (2.7 ± 1.9 min/day; *p* = 0.14), sedentary time (−10.9 ± 11.3 min/day; *p* = 0.34), and skeletal muscle work efficiency at 10 W (0.4 ± 0.4%; *p* = 0.37), 25 W (0.3 ± 0.6%; *p* = 0.6) and 50 W (−0.1 ± 0.4%; *p* = 0.79) were all not significantly different when compared with the post-weight loss baseline. Nevertheless, we cannot rule out diet differences in other components of TEE that were not quantified by Ebbeling et al., such as the thermic effect of feeding and sleeping metabolic rate.

## Reported diet differences were inflated by inclusion of subjects with implausible unaccounted energy

Although Ebbeling et al. provided the subjects with all their food to maintain a stable reduced body weight, the reported energy intake was 460 ± 46 kcal/day (*p* < 0.0001) less than the reported TEE. Therefore, it can be hypothesized that many subjects were likely consuming substantial quantities of nonstudy foods. In addition, large within-subject fluctuations in TEE during the weight loss maintenance period were often unaccompanied by weight changes or changes in reported energy intake to offset the imbalance due to TEE changes. Adjusting for changes in body energy stores (calculated using an assumed energy equivalent of 7700 kcal per kg of weight change), the mean amount of unaccounted energy was 483 ± 46 kcal/day (*p* < 0.0001).

When calculated according to the revised analysis plan, the substantial TEE diet effect depended on including subjects with excessive amounts of unaccounted energy. Figure 1b illustrates the diminution of the TEE diet effect when increasingly stringent thresholds were used to remove subjects with excessive unaccounted energy (*r* = 0.94; *p* < 0.0001). A statistically significant TEE diet effect (*p* < 0.05) required inclusion of subjects with >600 kcal/day of unaccounted energy. The intercept of the best-fit line was 89 kcal/day indicating that when the data were adjusted to be commensurate with the law of energy conservation the TEE difference between low- and high- carbohydrate diets was not statistically significant and was approximately half of that reported by Ebbeling et al.

## Exploratory ANCOVA analysis

As an exploratory analysis, we abandoned the prerandomization TEE anchor point by using analysis of covariance (ANCOVA) during the weight loss maintenance period with age, sex, height, and body weight as covariates. Figure 1c, d show TEE adjusted for age, sex, and height plotted against body weight at the midpoint and end of the study, respectively. ANCOVA estimated that TEE was 255 ± 88 kcal/day (*p* = 0.004) greater on the low- versus high-carbohydrate diet at the study midpoint and 224 ± 81 kcal/day (*p* = 0.007) greater at the end, respectively. However, TEE was not significantly different between the moderate- versus high-carbohydrate diet at the midpoint (41 ± 90 kcal/day; *p* = 0.65) or at the end (60 ± 83 kcal/day; *p* = 0.47).

## Daily average CO_2_ production was not different between diets regardless of analysis plan

The DLW method provides an indirect measurement of daily average CO_2_ production. The greater the TEE, the greater the CO_2_ produced. Translating CO_2_ production into TEE requires an assumption about the ratio of CO_2_ production to O_2_ consumption—the daily respiratory quotient (RQ) which varies depending on the macronutrient mixture being oxidized [9]. Inaccurate RQ assumptions can result in a systematic bias in the TEE calculations [9].

Using the TEE data along with the RQ assumptions of Ebbeling et al., we back-calculated each subject’s CO_2_ production. No significant diet differences in CO_2_ production were found regardless of prerandomization anchor point (Fig. 1e) or ANCOVA (Fig. 1f). Therefore, TEE diet differences were entirely due to assumed RQ differences between the diets [9]. The larger the RQ differences, the larger the calculated differences in TEE.

Ebbeling et al. assumed the RQ difference between high- and low-carbohydrate diets was at the theoretical maximum value of 0.11 given that subjects consumed all study foods and any nonstudy foods were identical in composition to the study diets. However, this is unlikely given the large discrepancies between reported energy intake and TEE along with the fact that consumption of many study foods was unsupervised and therefore may not have been completely eaten. Any documentation of off-study foods has not been made available, but if such documentation exists it must rely on subjective self-reported food intake measurements that are known to be inaccurate and essentially worthless for quantitative assessment of energy balance [10].

Previous studies employing isocaloric diets varying in proportion of carbohydrate to fat to a greater degree than the low- and high-carbohydrate diets of Ebbeling et al. resulted in daily RQ differences of less than 0.1 when directly measured using respiratory chambers in subjects admitted to metabolic wards to ensure strict diet adherence [11, 12]. Therefore, the RQ assumptions of Ebbeling et al. likely overestimated the TEE differences between the low- and high-carbohydrate diets.

Figure 1g illustrates that the TEE diet effect reported by Ebbeling et al. rapidly decreases with the assumed differences in daily RQ between high- and low-carbohydrate diets. For example, if the RQ values were 0.88 and 0.81 (rather than 0.9 and 0.79 assumed by Ebbeling et al.) on high- and low-carbohydrate diets, respectively, then the TEE diet effect amounted to only ~100 kcal/day and was no longer statistically significant. Similar results were obtained using ANCOVA (Fig. 1h).

## Conclusion

When the data of Ebbeling et al. were reanalyzed according to the original preregistered analysis plan, the effects of low-carbohydrate diets on TEE were not significant. Reported diet differences in TEE using the revised analysis plan of Ebbeling et al. required inclusion of subjects with large amounts of unaccounted energy due to consumption of off-study food as well as large fluctuations in TEE that were uncorroborated by weight changes. An exploratory analysis using ANCOVA with the postrandomization data confirmed that diet influenced TEE when using the assumed RQ values of Ebbeling et al. However, regardless of analysis plan, the DLW data demonstrated no significant diet differences in daily CO_2_ production demonstrating that TEE diet differences depend entirely on the assumed RQ values that may be unrealistic. In the absence of objective data about off-study food intake attesting to the validity of the RQ assumptions, the data of Ebbeling et al. do not provide strong evidence to refute the null hypothesis that TEE is unaffected by varying the proportion of dietary carbohydrate to fat.
